# The Teeth and Hair: Their Homology and Pathological Intimacy

**Published:** 1893-11

**Authors:** S. H. Guilford

**Affiliations:** Philadelphia


### THE TEETH AND HAIR: THEIR HOMOLOGY AND PATHOLOGICAL INTIMACY.[1]

BY DR. S. H. GUILFORD, PHILADELPHIA.

Two opposite conditions are of especial interest to the scientific mind: one, the highest development of the individual; the other, some variation from the normal type. The first illustrates the perfect working of laws when not antagonized by adverse conditions, and the second how a variation may result from some slight disturbance of their operation.

One condition is as instructive as the other, for it is often only through the consideration of an abnormity that we are led to appreciate the harmony of co-ordinate parts and their adaptation to function.

Perhaps no organs of the human body exhibit such a variety of aberration from the normal type and number as the teeth, and this fact further furnishes a field for observation and study. The subject is broadened and the interest intensified, when we consider the

---

[1] Read before the World's Columbian Dental Congress, Chicago, August, 1893.

teeth in relation to other organs or structures with which they are intimately associated.

Of the epithelial products, the hair, next to the teeth, is the one that is most frequently found to be abnormally affected; and the fact that these two structures have often been found to be conjointly influenced, long ago attracted the attention of scientific observers, but seems never to have received the careful investigation that its importance demands.

In approaching this subject it will be necessary to first briefly consider the origin of each of these structures. The ovum of the vertebrates consists of a mass of protoplasmic matter contained in a connective-tissue envelope. By a process of segmentation the ovum produces a vast number of cells that form a membrane known as the blastoderm, and this eventually is resolved into three layers, respectively denominated the epiblast, mesoblast, and hypoblast. "From the epiblastic or upper layer are formed the epiderm or cuticle of the skin and all its appendages, such as the hair, nails, and enamel of the teeth; also the brain and nerves. From the mesoblastic or middle layer are formed the true skin, cartilage, bones, muscles, dentine, and cementum of the teeth, etc. From the hypoblastic layer are formed the epithelium of the mucous membrane and the various glands of the alimentary canal." (Cryer.)

Hairs are a product, therefore, of the epiblast, and are developed inside of pouches or sacs from the infant cells of the rete Malpighii which dip into the underlying corium. As they develop they push their way to the surface, attain their normal length, are shed, and again replaced by others. They are nourished by a formative organ known as the follicle.

Hair of varying length and fineness covers the entire external surface of the human body, with the exception of the soles of the feet, palms of the hands, the eyelids, the last phalanges of the fingers, and certain portions of the genital organs. Its greatest length is attained on the head, especially of the female, upon the cheeks, chin, and upper lips of the male, and in the pubic and axillary regions of both sexes. Darwin says, "Hair is first developed in human fœtuses at about the third or fourth month, when it appears on the eyebrows and face, and especially round the mouth, where it is much longer than on the head." At this early period of intra-uterine life no difference is noticeable between the male and the female fœtus in regard to the abundance or location of this growth, whereas in early childhood, and especially after puberty, the difference between the sexes in this respect is very apparent.

The downy growth of lanugo with which the human fœtus is covered is shed just before or at birth, and is succeeded by a less vigorous growth, which in the normal individual continues through life. This growth under peculiar abnormal conditions may be entirely lacking, or it may, in the case of "sports" or "freaks," attain an unusual length, owing to an excessive vitality of the follicles.

We believe all biologists agree that mammalian teeth, if not indeed the teeth of all the vertebrata, are developed in a sac or a pouch formed in part and in its earlier stages by a dipping down or invagination of the oral epithelium, which in time becomes the enamel-organ. That under this depressed epithelial layer a papilla arises from the corium beneath which the dentine is formed; the said papilla being eventually known as the dental pulp. The cementum is formed from what is known as the dental sacculus, a specialized product of the connective tissue which encloses both the enamel and dental organs, completely surrounding them.

Such being the origin of the tooth and its different tissues, are we correct in calling the mammalian tooth a "dermal appendage"? The close relationship existing between the teeth of certain vertebrates and their dermal armature or scales is best seen in the shark, dog-fish, and other elasmobranch fishes.

Of the placoid scale of the shark, Greenbaur says, "The placoid scale has the structure of the dentine, is covered with enamel, and is continued at its base into a plate formed of osseous tissue; as they agree with the teeth in structure, they may be spoken of as dermal denticles."

Some of our best-known anatomists and physiologists consider the teeth as "specialized dermal appendages," which is accepted by biologists as reasonably conclusive. Each of the various blastodermic layers gives origin to a variety of tissues. Is it therefore not reasonable to suppose that where one tissue of a certain layer is pathologically affected, others of the same layer may also be? and if in a number of instances such is shown to be the case, is it not evident that all of these tissues have the same origin?

Instances are not wanting among the various species of vertebrata, where an abnormity in one product of the epiblastic layer is accompanied by a corresponding one in another of the same layer. We know that in the order of Edentata, in which some of the individuals are without teeth and others are lacking the normal number of their class, their integuments are also very variable in character.

In the hairless dogs of China and Japan the dental system is said to be greatly reduced, as compared with others of their class who have a normal hairy covering. The manatee, or sea-cow, which is an herbivorous aquatic animal, having stiff hairs or bristles sparsely scattered over its skin, has a most peculiar and imperfect dentition, differing from any other species of mammalia.

Ascending the scale to man, we find many evidences of the same relationship between products of the epiblastic layer. In albinism the chief peculiarities are lack of coloring-matter in the skin and hair, and a pink iris. Both of these tissues are epiblastic products, and each is abnormally affected.

Within the past year the writer had the privilege of examining a family of hairless people from the interior of France, consisting of mother, daughter, and son. In each individual there was no hair upon the scalp, the fine down or lanugo usually covering the body was lacking, and the finger-nails were thick, narrow, and pointed, resembling the talons of a bird. The teeth, however, were normal in size, form, and number. Wilson mentions the case of a woman, aged thirty-three years, whose entire body was covered with thick and long hair and who had never perspired. The abnormal growth of hair was not congenital, but began at puberty and remained. The same author speaks of the nails frequently showing evidences of abnormity in connection with either absence or superabundance of hair.

Dr. E. P. Bradbury, of Boston, reports the case of a man, aged twenty-four, who was edentulous and claimed never to have erupted any teeth. This peculiarity was accompanied by an entire absence of saliva. His tongue was dry and leathery, and his speech thick. He had never been able to take solid food of any kind, but subsisted on soups and soft food.

Coming now to the correlation of the hair and teeth, let us consider cases in which an abnormity of one tissue is accompanied by abnormity in the other. In some instances deficiency of one product is accompanied by deficiency in the other, whereas in others deficiency of one is associated with redundancy of the other. Of deficiency of both structures, the most notable instance on record, and perhaps the only one of its character, is that of a man whom the writer exhibited before certain medical and dental societies in Philadelphia in 1883, a full account of which, with genealogy and verification, may be found in the *Dental Cosmos* for March, 1883.

The individual was forty-eight years of age, and had never had any teeth, either temporary or permanent. His head was nearly bald, being only slightly covered with fine down; and while hair was present in the pubic and axillary regions, the surface of his body was entirely lacking in the surface-hairs and lanugo usually present. Owing either to the absence or suppression of the sudoriparous glands, he had never perspired. In addition to these peculiarities, he had no sense of smell and very little sense of taste, a combination of peculiarities which literally constitute him *sui generis.* He is still living and in good health, and of his six children only two (girls) show any signs of inherited abnormity, and then only in having about half the usual number of teeth.

Another case of somewhat similar character, but much less marked, is reported by M. Jarré, of Paris, a translation of which appears in the *Dental Cosmos* for June, 1892. The subject was a boy, twelve years of age, whose scalp had a scant covering of hair and whose body was entirely free from surface-hairs. His lower jaw was edentulous, and the upper jaw contained but five teeth. The finger-nails also presented an abnormal appearance, being covered with white spots and streaks.

Of the second class, in which deficiency or abnormity of the teeth or jaws is associated with excessive development of the hair, there are several notable instances on record. One of these is the well-known case of Julia Pastrana, a Spanish ballet-dancer, who appeared in Europe many years ago. The hair upon her head was dark, coarse, and strong, while upon her cheeks and chin she wore a full beard several inches long. Her dentition was normal, as testified to by both M. Magitot and Sir John Tomes, who examined her mouth, but the alveolar and overlying soft tissues were hypertrophied to such an extent that only the coronal surfaces of the teeth were visible.

Another case, mentioned by Parreidt, is that of Krao, an Indian girl seven years of age, who was exhibited in Europe some years ago. Her body was covered with a well-developed growth of hair, and her jaws presented an hypertrophied condition, her teeth being normal in number and form. A more remarkable example, in which an abnormal growth of hair was associated with a deficiency of teeth, is that of Fedor Jeftichejew, the so-called dog-faced man, who has been exhibited the world over, and whom your essayist had the privilege of examining less than a year ago. In his lower jaw there are three teeth, one cuspid and two incisors. In the upper jaw there are only two cuspids; one tooth, a lower incisor, having been extracted.

When first exhibited, Fedor was about three years old, and was accompanied by his father Audrian, who possessed the same peculiarities, but who has since died. Fedor, now a man twenty-three years of age, has his entire body covered with a fair growth of hair, while upon his face, including the nose and forehead, there is a strong growth of soft hair several inches in length. In his case also the sudoriparous glands seem to be poorly developed, for he perspires but little. Still another case of excessive hair-development, associated with a more or less defective development of the dental organs, is found in the "Burmese Hairy Family" exhibited at various times in this country and Europe. Three members of this family, the grandfather, the mother, and Moung Phoset, the son, had their faces and bodies covered with long dark hair of a silky character. Their hands were not thus covered, but upon their foreheads, cheeks, noses, and ears the growth was excessively abundant.

That upon the forehead of Moung Phoset was so long that it was parted in the middle and carried back and secured behind the head. The grandfather died in Ava, and was never on exhibition, but was seen and reported upon by Captain Crawford, who states that "the hair on the face, ears, and nose was eight inches long, and on the breast and shoulders four or five inches."

As to the lack of teeth in these cases, writers differ in their accounts. Crawford says the mother lacked the cuspids and molars in each jaw; a later writer speaks of her as lacking many of the teeth, but as a matter of fact, Dr. John A. Daly, of Washington, D. C., in 1888, removed no less than fifteen teeth from her jaws.

Moung Phoset, the son, is reported as lacking many of his teeth, but this could neither be verified nor contradicted, inasmuch as the exhibitor in charge invariably refused to allow his mouth to be examined.

From the seven cases recited we see an absence or scanty covering of hair on the scalp, with entire absence of surface-hairs on the body. With this in two cases there was deformity of the nails, and in two cases partial or entire absence of sudoriparous glands, and in one case a total lack of the sense of smell and an imperfect sense of taste.

In the four cases of excessive hair development we find in two cases the normal number of teeth, with hypertrophy of the alveolar process and gum-tissues; in one case we find only six teeth *in situ*, while in the last we have no true record of the dental organs.

But there is another source from which we can derive evidence of the intimate relation of these tissues, and one seldom alluded to, that of ovarian cysts. In these aberrations of structure, which Wilson speaks of as "normal tissues abnormally placed," there are usually found hair, teeth, alveolar tissue, and sometimes nails; all products of the epiblastic and mesoblastic layers.

Professor Roswell Park says, "Such products arise from isolated portions of the epiblast or mesoblast, or both, which during the development of the embryo have been displaced and located somewhere where they do not properly belong." Such islands of tissue retain nearly all their embryonal possibilities, and, given an impetus, develop into any or all of the tissues which they might normally produce.

In view of these facts, the question naturally suggests itself, "Why should these two particular products of the epiderm so often be found to be conjointly affected?" A satisfactory answer has never been and cannot now be given, but the consideration of two points may shed some little light on the subject.

*First.* The development of the hair and teeth in the human embryo are more nearly contemporaneous than that of the other epidermoid tissues, the first covering of hair or lanugo being noticeable about the fourth month of fœtal life, and the formation of the dentine cap or calcification occurring, according to Magitot, at the same period.

*Second.* The homology of the two structures is strikingly illustrated (*a*) in their being developed within a sac formed by the dipping down and infolding of the epithelium; (*b*) in their being first formed and afterwards nourished by a papilla or follicle; (*c*) in their limitation of growth; (*d*) in their being shed and replaced, once or oftener, by structures of similar character; and (*e*) in being so commonly found associated in dermoid cysts. We are therefore justified in concluding:

*First.* That where one product of the epithelial layer is abnormally affected, one or more of the other products of the same layer are also apt to be.

*Second.* That the two most commonly affected are the hair and the teeth.

*Third.* That this is shown by numerous examples throughout the vertebrata, including man.

*Fourth.* That the intimacy between these two products is probably due to the contemporaneousness of their inception, as well as to many points of similarity in their character and growth.

*Fifth.* That when thus co-ordinately affected, the manifestations are not uniform but variable.

*Sixth.* That this variability cannot be accounted for in the light of present knowledge.

